# Hydrothermal Hot Isostatic Pressing (HHIP)—Experimental Proof of Concept

**DOI:** 10.3390/ma17112716

**Published:** 2024-06-03

**Authors:** Yaron Aviezer, Shmuel Ariely, Menachem Bamberger, Denis Zolotaryov, Shai Essel, Ori Lahav

**Affiliations:** 1 Faculty of Civil and Environmental Engineering, Technion—Israel Institute of Technology, Haifa 32000, Israel; aviezery@cv.technion.ac.il (Y.A.); agori@technion.ac.il (O.L.); 2 Israel Institute of Materials Manufacturing Technologies, Technion—Israel Institute of Technology, Haifa 32000, Israel; ashmuel@technion.ac.il (S.A.); denisz@trdf.technion.ac.il (D.Z.); 3 Department of Materials Science and Engineering, Technion—Israel Institute of Technology, Haifa 32000, Israel; 4 Faculty of Mechanical Engineering, Technion—Israel Institute of Technology, Haifa 32000, Israel; shaie@campus.technion.ac.il

**Keywords:** HHIP, AlSi10Mg, hydrothermal water conditions, additive manufacturing

## Abstract

A new hydrothermal hot isostatic pressing (HHIP) approach, involving hydrothermal water conditions and no usage of inert gas, was hypothesized and tested on 3D-printed Al-10%Si-0.3%Mg (%Wt) parts. The aluminum-based metal was practically inert at the applied HHIPing conditions of 300–350 MPa and 250–350 °C, which enabled the employment of a long (6–24 h) HHIP treatment with hardly any loss of material (the overall loss due to corrosion was mostly <0.5% *w*/*w*). Applying the new approach on the above-mentioned samples resulted in an 85.7% reduction in the AM micro-pores, along with a 90.8% reduction in the pores’ surface area at a temperature of 350 °C, which is much lower than the 500–520 °C applied in common argon-based aluminum HIPing treatments, while practically maintaining the as-recieved microstructure. These results show that better mechanical properties can be expected when using the suggested treatment without affecting the material fatigue resistance due to grain growth. The proof of concept presented in this work can pave the way to applying the new HHIPing approach to other AM metal parts.

## 1. Introduction

Hydrothermal conditions can be used to expose solid metals to unique high-temperature and pressure conditions. In the industrial manufacturing of materials, a set of methods combining pressure and heat are commonly implemented to improve the product’s quality following casting or 3D printing [1]. The pressure/heat combination induces the diffusivity and hence the closure of voids and defects in the material; by doing so, the samples’ mechanical properties improve. In metals, such porosities and voids are found in castings, and more pertinently, in additively manufactured metal parts [2,3]. 

Hot isostatic pressing (HIP) is a well-established method for healing internal pores in cast parts and is widely used to significantly increase the fatigue life in critical components. Applying HIP to AM AlSi10Mg, for example, is carried out at temperatures of ~500 °C and a pressure of 100 MPa. Such conditions can close/heal internal pores and a lack of fusion defects, thus resulting in an increase in the fatigue strength [4] and in improved material ductility; however, this reduces the strength [5]. 

At the reduced yield stress and the higher diffusion rates associated with high temperatures, the applied pressure may lead to pore collapse via small-scale plastic flow and material transport, which, under ideal conditions, also bonds the pore interface [6]. In aluminum, although there are standards for the HIP of aluminum alloy casting [7] and for AlSi10Mg AM manufactured parts [8], some researchers do not recommend that the HIP treatment is applied as it may destroy the mechanical properties due to grain growth and other unwanted harmful thermal treatment effects, as recently concluded, for example, by Hirata et al. (2020) [9], who investigated the applicability of HIP in eliminating the internal pores in selective laser-melted (SLM) AlSi10Mg. These authors recommended optimizing the SLM irradiation conditions in the fabrication of SLM aluminum alloys, rather than using HIP. 

The HIP conditions are generally chosen such that the gas pressure is greater than the reduced yield point, up to the point of approaching the partial melting of the material [10] at the HIPing temperature. Plastic flow can then occur on a microscopic scale. Under HIP conditions, considerable particle shear occurs, and creep processes such as diffusion through grain interiors, diffusion around grain boundaries and dislocation creep occur at relatively high rates. These are the processes that are primarily responsible for the densification of the subjected parts [6].

In the commonly applied HIP practice, inert gas, such as argon, is compressed inside a furnace to allow simultaneous heating and pressing of the part. 

The main limitations of the current HIP practice are (1) Limited pressure: the commonly used gas-compressed HIP systems work in the pressure range of 100–200 MPa [6], which dictates a high working temperature, e.g., [11,12]; (2) High temperatures: under a limited gas pressure environment, the sample’s temperature is increased to allow efficient diffusion. Concurrently, the high temperatures often reduce the metals’ mechanical properties owing to the grain growth effect, see, e.g., [13]; and (3) Costs: a high operating temperature increases the energy costs due to a larger heat loss during the HIP holding time [14], followed by the coupling of high pressure and temperature, which leads to expensive infrastructure and operation costs. 

Cold Isostatic pressing (CIP) is also common practice. In this procedure, a pressed liquid (usually oil) isostatically presses a body to increase its density. However, these methods are usually applied for ceramic green bodies, e.g., in [15]. A hydrostatic CIP treatment was recently evaluated by Cuesta et al. (2019) [16] for additively manufactured ANSI 316L metallic components, with contradictory results.

The use of water at a high pressure and temperature for the hydrothermal hot isostatic pressing of mica powders was previously proposed and investigated, e.g., by [17,18]. However, to the best of our knowledge, the hydrothermal isostatic pressing treatment of metal parts, and more specifically for additive material metal parts, has not been attempted.

The current proof of concept (PoC) aims to utilize a unique hydrothermal application (H_2_O at hydrothermal conditions) to develop a new cost-effective HIP metal treatment concept. The work described here focused on the reduction in internal pores in AM aluminum samples while minimizing the potential corrosion reactions in the prevailing hydrothermal environment. Aluminum alloys were selected for the PoC stage because of their low melting temperature. The “regular” argon HIP treatment for aluminum and its alloys is performed at 500 °C/100 MPa [6,9], while the proposed hydrothermal HIP was performed in the 250–350 °C/300–350 MPa range. 

The hydrothermal HIP (termed HHIP) treatment proposed herein is based on feeding water to the HIP chamber and then raising it to the required operating pressure and temperature by a high-pressure water pump and an electrical heater. The proposed treatment has the potential to dramatically decrease the process temperature and the retention time of the procedure, owing to the much higher applied pressure. This has the potential to open a new branch of isostatic pressure treatments, with a decreased grain growth (which tempers the mechanical properties) due to the lower time and temperature exposure. Furthermore, the water media is safer, cheaper, and more environmentally friendly than the pressed inert gas solution currently applied in state-of-the-art HIP systems. However, the presence of an aqueous environment at elevated temperatures introduces questions regarding the chemical compatibility and possible corrosion of the treated metallic parts. In a recent work, Trowell et al. (2022) [19,20] investigated the use of metal slugs and plates for hydrogen production in supercritical water (P = 23 MPa, T = 425–655 K). They observed full hydrogen yields with aluminum, magnesium, and zinc samples in 30 min, while silicon and zirconium showed poor reactivity, and titanium showed no reactivity at all in the water medium. With respect to aluminum, the possible reactions involving the oxidation of aluminum and reduction of H_2_O are as follows:

(1) $$2Al+6H_{2}O\to 3H_{2}+2Al(OH{)}_{3}$$

(2) $$2Al+4H_{2}O\to 3H_{2}+2AlO\left(OH\right)$$

(3) $$2Al+3H_{2}O\to 3H_{2}+Al_{2}O_{3}$$

A link between the oxide species’ solubility and its reactivity with water was proposed by [21]. Cook et al. (2012) [22] presented Pourbaix diagrams of a few metals under subcritical (T = 350 °C) and supercritical (T = 400 °C) water environments, both at a pressure of 25 MPa. For aluminum, it was reported that a minimum concentration of 10^−8^ mol/kg of dissolved aluminum is required to maintain a passive oxide layer. However, the physical and chemical characteristics of the water vary considerably in the literature between the data presented at 400 °C/25 MPa and the 250–350 °C/250–350 MPa working conditions proposed in the current work. Thus, although preliminary equilibrium aluminum stability calculations using the PhreeQC aqueous speciation modelling tool [23] indicated a stable Al_2_O_3_ phase formation, to verify this, critical aluminum corrosion tests were carried out, at the proposed hydrothermal working conditions. 

## 2. Experimental

### 2.1. Materials

Three aluminum alloys were selected for the PoC experiments. Two commercial alloys (Al6061/2024) were used for the screening corrosion experiment under hydrothermal conditions, and AlSi10Mg was selected for the AM HIP experiments. The AM samples were stress relieved at 300 °C for 120 min prior to removal from the SLM base plate. The composition of the selected alloys is shown in Table 1. Deionized water (DIW) at EC = 0.5–1 µS/cm was used in all the experiments. The Al6061/2024 samples were made of 1 mm thick plate strip cuts. The AlSi10Mg AM sample was a 3 mm ID/ca. 20 mm cylinder, produced by the EOS M290 3D printer (EOS GmbH, Karailling, Germany) using 20–75 µm raw material particles, after common-practice stress relief.

### 2.2. Experimental System

**Figure 1:**
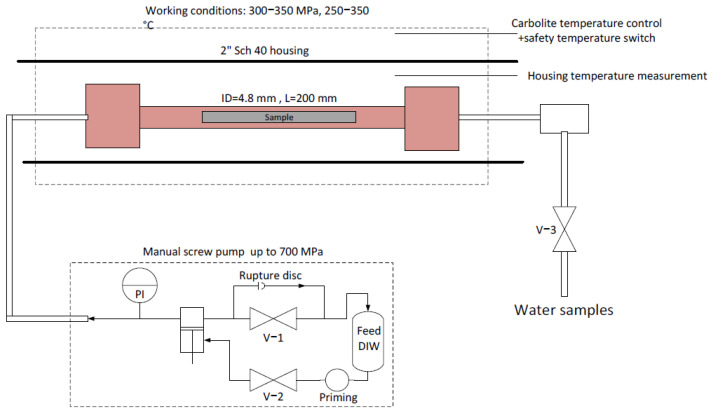
Experimental hydrothermal HIPing system.

The experimental system is shown schematically in Figure 1. The required pressure was created using a manual high-pressure screw piston, manufactured by Sitec High-Pressure Technology AG (Zurich, Switzerland). All piping and fittings were supplied by the same manufacturer. Heating was applied using a horizontal Carbolite 2″ furnace (Carbolite Gero, Hope Valley, UK), with a heating capacity of up to 1500 ± 1 °C. The HIPing reactor was a 9/16″ 200 mm SS316Ti spool rated for 400 MPa with an internal diameter of 4.8 mm. Water samples were extracted via valve V −3.

### 2.3. Hydrothermal Experiments

A set of ten PoC hydrothermal experiments were divided into preliminary aluminum compatibility corrosion tests, which aimed to verify the low reactivity of the aluminum alloys, and hydrothermal HIP experiments for the AM AlSi10Mg samples. The possible working conditions were limited by the design pressure of the test reactor (400 MPa). Hence, the working conditions varied between temperatures of 250, 300, and 350 °C, and pressures of 300 and 350 MPa. The treatment duration was either 6–8 h or 24 h. The complete experimental setup is shown in Table 2, with the relevant water analysis results. During the heating step and the corresponding water expansion, the system was regulated to attain the required pressure by adjusting the manual high-pressure screw pump.

### 2.4. Analyses

The inorganic species composition of the water samples was determined using a Thermo Fisher Scientific ICP-OES (Thermo Fisher Scientific, Cambridge, UK). This was performed for the various aluminum alloys constituents, as well as for the HIP reactor and piping SS316Ti alloying species. The concentrations of dissolved Al, Cr, Cu, Fe, Mo, Ma, Ni, P, Si, Ti, S, Mg were quantified in the prewash solution, the solution inside the reactor and the final wash solution that followed each of the experiments. 

To evaluate the corrosion of the non-AM samples (Al2024 and Al6061) and the AM AlSi10Mg samples before and after the hydrothermal treatment, the samples were embedded in conductive Bakelite, ground using 220 MESH SiC paper, polished using the diamond suspension, and etched with colloidal silica. Their cross-sections were evaluated by Energy-Dispersive X-ray Spectroscopy (EDS) analysis, performed using the FEI Inspect S50 microscope (FEI, Moravia, Czech Republic). The system’s EDS detector was INCA Penta FETx3 (Oxford Instruments, Belfast, UK).

Measuring the pore sizes and distribution in the AM AlSi10Mg samples was performed using EasyTOM XL, a flexible X-ray micro and ultra tomography system (RX Solutions, Chavanod, France). The results were evaluated using Xact and VGStudio max 3.50 tools. A voxel size of 3.5 µm, 2625 slices, 110 kV, and 80 µA were the main analysis setup parameters. 

The hardness of the samples was measured using the Future-Tech Microhardness Tester, FM-110 (Future-Tech Corp., Kanagawa, Japan).

Phase analysis was performed using a Rigaku SmartLab X-ray diffractometer, which operated in a continuous scanning mode with CuKα radiation. The microstructure, local phase identification, distribution, and local composition were studied using a Zeiss Ultra-Plus FEG-SEM equipped with an Oxford EDS detector and Bruker EBSD detector. The microstructure was captured using a high-definition back-scattered electron detector.

## 3. Results and Discussion

### 3.1. Corrosion 

#### 3.1.1. Experimental Conditions and the Resulting Species in the Water Samples 

Table 2 lists the experimental conditions and the ICP results of the main dissolved species encountered in the water samples in the various tests. The reactor volume was 3.62 mL. The weight change calculation in each sample is based on the samples’ weight, thereby including the added weight of the oxide corrosion layer. The weight loss calculation is based on the aqueous concentration of the Mg^2+^ (which is absent in the reactor and piping metal), considering its alloying composition in the tested samples. 

The results shown in Table 2 indicate, based on the samples’ weights and the aqueous metal concentrations, that the overall corrosion of the part samples was in most cases less than 0.5% *w*/*w* during the HIP treatment timeframe. Furthermore, in all the aqueous samples, titanium ions, which may stem from the corrosion of the reactor wall, were not detected at all. 

#### 3.1.2. Inspection of the Surface Corrosion of the Al 6061/2024 Samples

**Figure 2:**
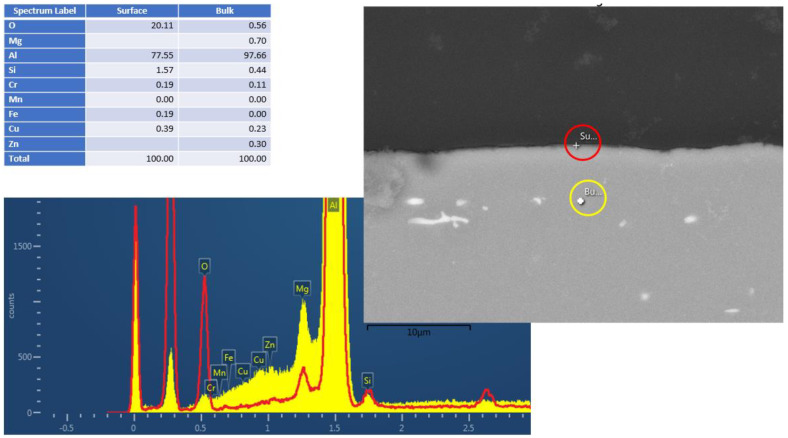
Al6061 cross–section EDS results after HHIP. Red and yellow are the t EDS spectra from surface and bulk material, respectively.

**Figure 3:**
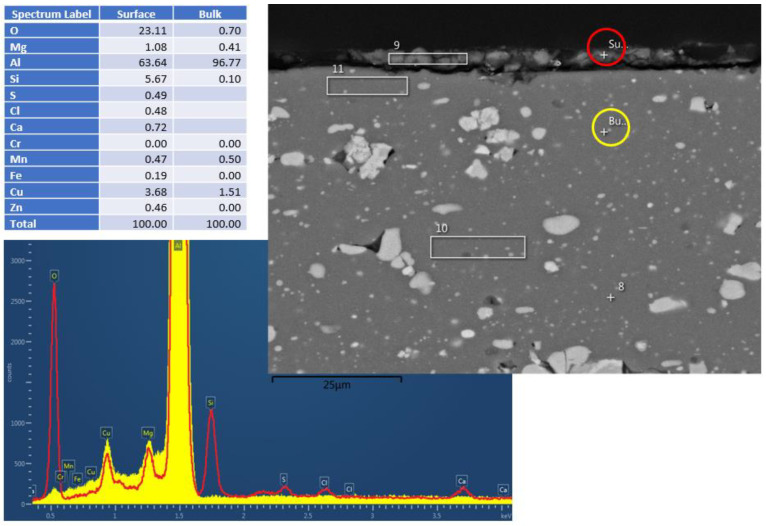
Al2024 cross–section EDS results after HHIP. Red and yellow are the spectra from surface and bulk material, respectively.

Figure 2 and Figure 3 show the cross-section EDS results of the Al6061 (Experiment 3) and Al2024 (Experiment 5), respectively, following the hydrothermal treatment. The figures consist of SEM pictures, indicating the bulk (yellow circle) and surface (red circle) spot locations of the EDS analysis. The elemental analysis results (in wt.%) are shown in Figure 2; these clearly indicate the surface oxygen and the relevant silicon, magnesium, and aluminum peaks. Similar analyses of the Al2024 pretreated sample (not presented) showed a 2.5 µm Al/O layer, whereas no oxide layer was visible in the Al6061 pretreated sample. As indicated in the results shown in Figure 2 and Figure 3, a very thin corrosion layer was visible in the Al 6061 post-treatment sample. Pointing the EDS exactly on the sample surface resulted in a minor oxygen peak.

That said, a certain corrosion layer was found during the EDS surface layer evaluation of the Al2024 sample (Experiment 5) after the hydrothermal treatment, as shown in Figure 3. Silicon and copper accumulation were detected within the corrosion layer, together with alumina (Al/O ratio close to Al_2_O_3_). 

#### 3.1.3. Inspection of the Surface Corrosion of AM AlSi10Mg Samples

**Figure 4:**
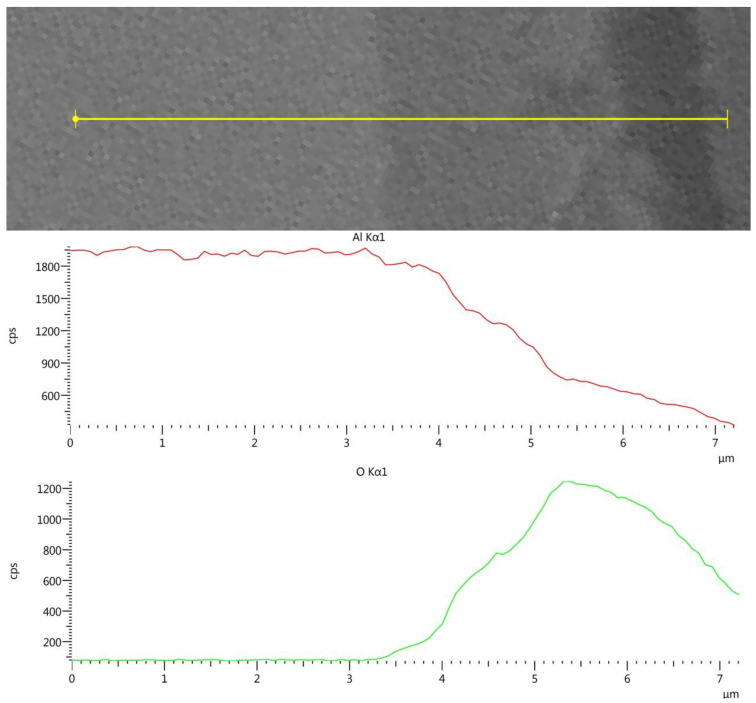
AM AlSi10Mg cross-section EDS line-scan results following HHIP.

Figure 4 shows the EDS line-scan results of the AlSi10Mg post-treatment sample (Experiment 10) across the surface. A similar analysis of the pretreated sample showed a 2.5 µm oxide layer.

Figure 4 shows a 3–3.5 µm Al/O layer. The results indicate that the surface oxide layer was increased by about 1 µm during the 24 h hydrothermal HIP treatment. However, the corrosion indications on the surfaces of the non-AM Al6061, Al2024 and AM AlSi10Mg post-treated samples are totally different from the fast dissolution of the aluminum in the 25 MPa/300–400 °C experiments reported by Trowell et al. [19,20,21]. The aluminum samples in the current research, which were exposed to hydrothermal conditions of 300–350 MPa/250–350 °C, showed minimal corrosion effects, which were consistent with the PhreeQC simulation.

### 3.2. Tomography Tests (MicroCT)

To examine the effectiveness of the hydrothermal HIP treatment for the closure of internal micropores, three AM AlSi10Mg treated samples (Experiments 8–10) were scanned against an untreated AM sample. Table 3 shows the four microCT sample results.

The results of the microCT tests indicate that the hydrothermal HIPing can reduce the volume of the pore by 85% at a temperature of 350 °C, which is much lower than the temperature applied in common aluminum HIPing. The cross-section of the pores (or “defects”) was reduced by more than 90%, which should manifest itself in better mechanical properties. A study of the high-temperature mechanical properties of the AM AlSi10Mg after HIPing at various conditions is pending. However, the high-temperature mechanical properties of the un-HIPed AM AlSi10Mg was previously studied by Uzan et al. (2018) [27]. According to this work, AM AlSi10Mg, an aluminum matrix reinforced by submicron Si particles, exhibited, for example, a creep parameter with a stress exponent of 25 ± 2, which is five times higher than the value of a pure aluminum alloy. This fact may explain the rather low value of pore closure at 250 °C, even at an applied pressure that was more than twice the value of the yield stress (YS) of this AM material. Furthermore, according to [27], the YS of AlSi10Mg at 350 °C is 30 MPa, i.e., much lower than 132 MPa at 250 °C, which may explain the high closure of pores in the current study (85.7%). Nevertheless, a much higher pressure (300 MPa, 10 times the YS) was required.

A preliminary hardness analysis of the small AM AlSi10Mg post-treated samples did not show any conclusive hardness change, as shown in Table 4. Further mechanical property analyses should be performed in future experiments.

### 3.3. Grain Size and the Microstructure of the AM AlSi10Mg Samples

**Figure 5:**
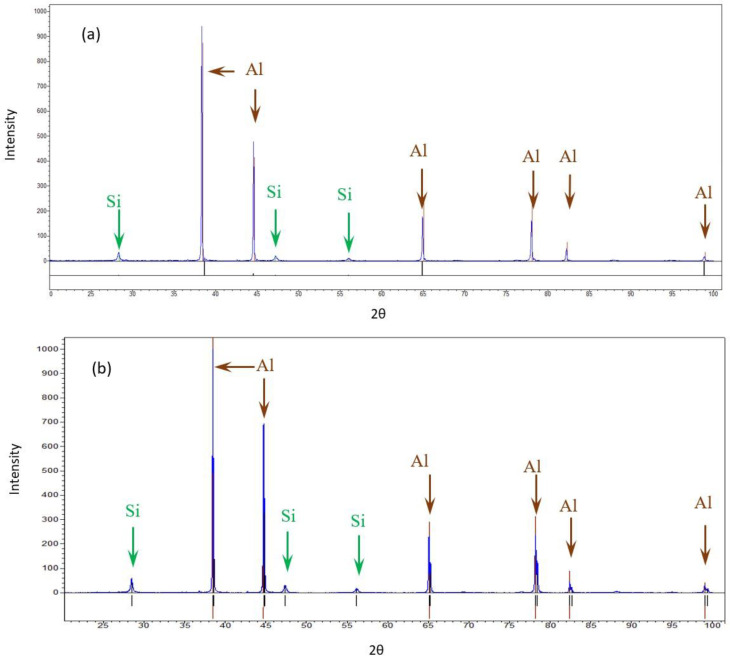
XRD diffractions of the AM Al10SiMg alloy. (**a**) Pretreated material (**b**) Post-treated material.

The bulk XRD measurements of the pretreated and post-treated (experiment 10) samples show that both samples consisted of Al and Si. The differences in the Al peak ratios between the pretreated sample and the post-treated sample may suggest differences in orientation due to the treatment. No similar differences were detected in the case of Si. No additional phase was detected, as shown in Figure 5.

**Figure 6:**
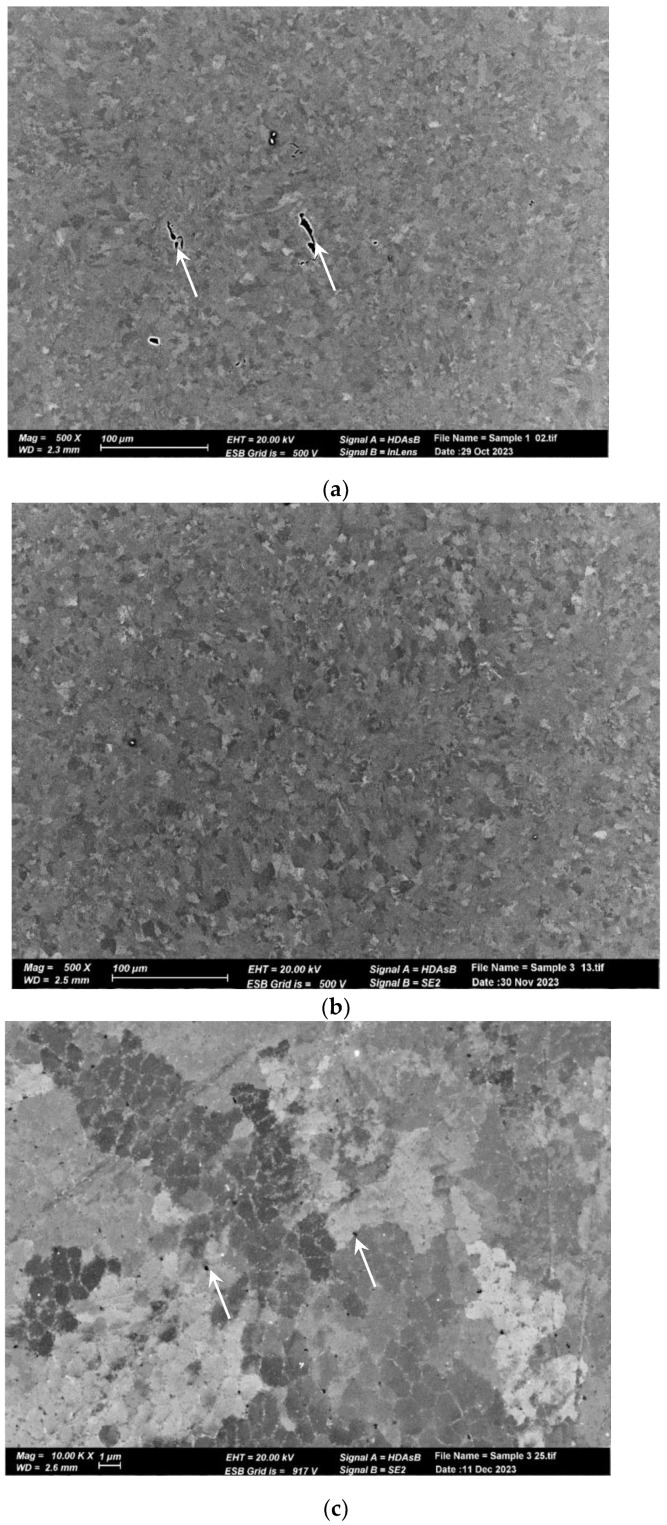
SEM micrographs of AM Al10SiMg alloy. (**a**) As-received structure. Note the pores aligned with the printing direction. (**b**) Treated material structure. No pores were noticed at a low magnification. (**c**) A high magnification (**b**) shows sub-micron round pores.

**Figure 7:**
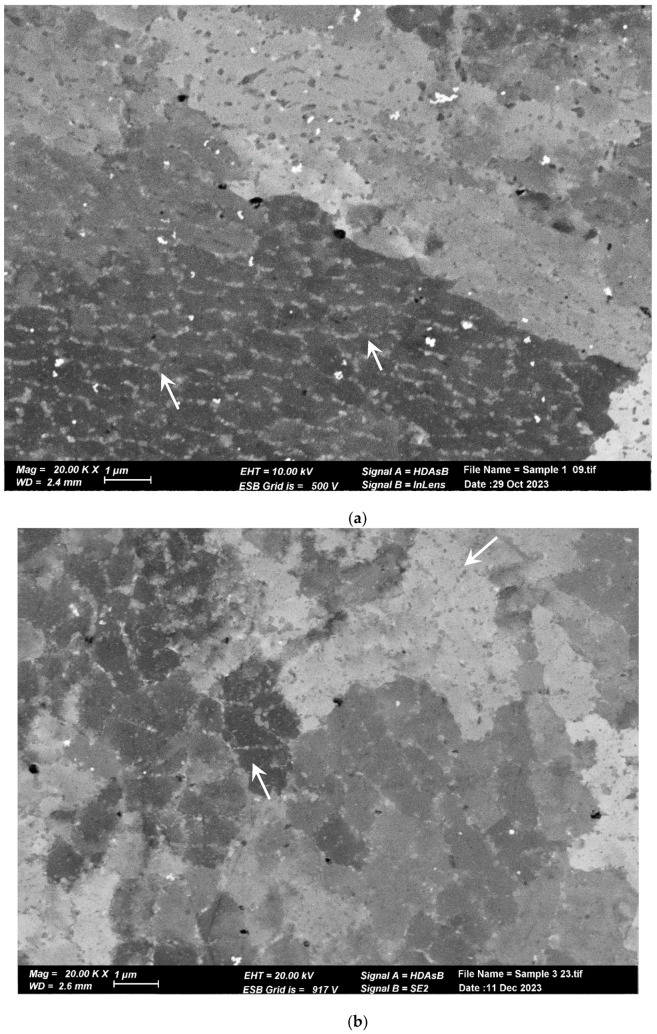
SEM image of cellular sub-structure. Each cell is decorated with Si nanoprecipitates. (**a**) Pretreated material. (**b**) Post-treated material. The cells and Si nanoprecipitates have the same size and shape in both materials.

**Figure 8:**
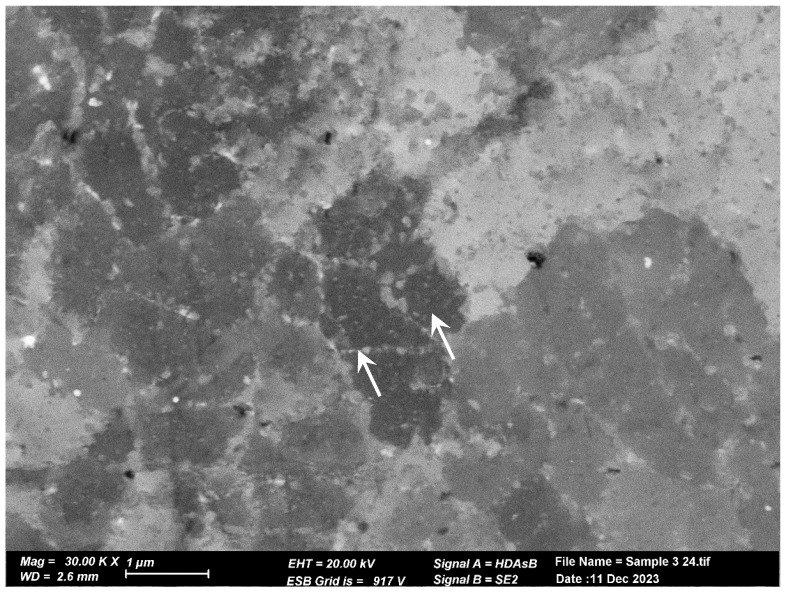
High magnification SEM image of the treated material. Tiny Si particles can be noted in the cells, as pointed out by the arrows.

The microstructure of the pretreated material, shown in Figure 6a, reveals elongated and aligned pores (indicated by the white arrows) along the printing direction. However, pores are not visible at the same magnification in the post-treated material microstructure (Figure 6b). Upon closer inspection of the post-treated material microstructure, sub-micron round pores (indicated by the white arrows) can be observed in Figure 6b,c. Figure 7 displays the microstructure of both samples, which comprise small grains and exhibit a fine cellular structure that is decorated with nanoparticles (indicated by the white arrows). The size of the cells is approximately 1 µm, while the particle size ranges from 200 to 400 nm. Examination with a higher magnification uncovered even smaller particles within these cells (indicated by the white arrows), as illustrated in Figure 8 for the pretreated material. The compositional measurements indicate that the nanoparticles decorating the cellular structure are Si. No eutectic structure was observed in either case.

**Figure 9:**
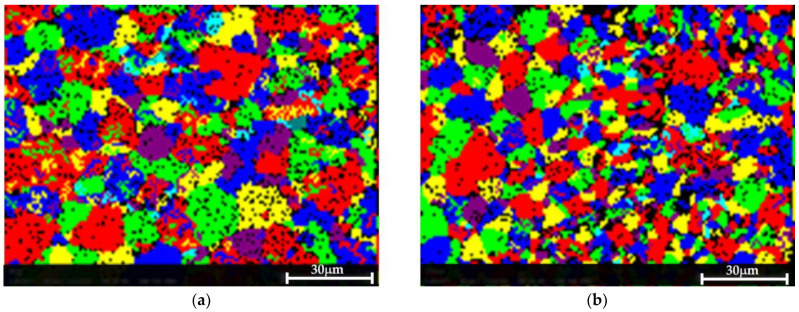
EBSD grain map showing that the treatment did not result in a consequential change in the grain size. (**a**) The pretreated sample, in which the measured average grain size was 8.2 µm. (**b**) The post-treated material, in which the average measured grain size was 9.5 µm.

Figure 9 shows the EBSD grain maps of both materials. Only a slight difference in the grain size after the treatment was detected: 8.2 µm in the pretreated material versus 9.5 µm in the post-treated sample.

**Figure 10:**
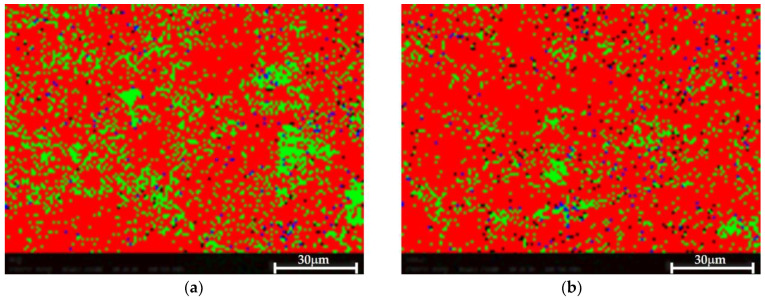
EBSD phase map. Al—red, Si—green, Mg_2_Si—blue. Very small Al and Mg_2_Si are detected in both samples. (**a**) Pretreated material, (**b**) Post-treated material.

The EBSD phase mapping demonstrates the presence of very small Si and Mg_2_Si dispersions in both cases, as shown in Figure 10. No significant difference was observed between both samples, suggesting that the proposed HHIP did not impact or impair the material’s microstructure.

**Figure 11:**
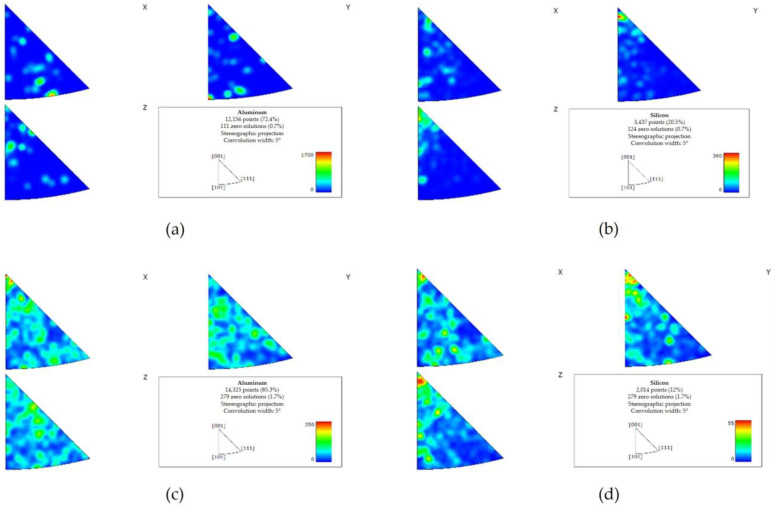
EBSD texture maps of the pretreated material (**a**,**b**) and the post-treated material (**c**,**d**). A preferred directionality of Al and Si was detected in the pretreated material ((**a**,**b**), respectively). Following the treatment, the preferred directionality was less significant (**c**) Al, (**d**) Si.

The EBSD texture analysis indicated a preferred directionality of Al and Si in the pretreated material (Figure 11a,b). Less significant directionality was observed in the post-treated material (Figure 11c,d).

The Al-Si phase diagram is a well-known eutectic diagram showing the minimal solubility of silicon (Si) in aluminum (Al). Adding a small amount of magnesium (Mg) further reduces the solubility of Si in Al. The Mg addition results in the formation of Mg_2_Si, but the formation of the Al-Si eutectic structure can still be expected.

Due to the high cooling rate characteristic of AM, a network eutectic structure is formed, which is composed of small aluminum grains, 8–9 μm in size [28]. These grains are made up of a network of silicon nanoparticles, each of which is approximately 100 nm in size, and the cells of this network are about 1 µm. Additionally, Mg_2_Si nanoparticles were detected between the Si nanoparticles. Within these cells, smaller Si particles were also found, measuring roughly 10 nm in size, as measured by SEM; this is shown in Figure 8. It is worth noting that the Mg_2_Si phase was not detected by XRD due to its relatively small content (around 1 wt.%), but the EBSD identified it. Upon conducting a comprehensive examination of the two studies, it was revealed that the alloy undergoes a eutectic cellular solidification process in the presence of silicon at the cell boundaries. The printing procedure involves multiple cycles of heating and cooling, which results in the annealing and fracturing of the silicon network, ultimately resulting in the creation of silicon particles at the nano-scale level.

Following the printing (and stress relief) processes, the material was subject to a HHIP treatment, leading to self-diffusion and plastic flow. Nevertheless, the energy level was insufficient for recrystallization and long-range diffusion, and did not affect the eutectic network structure. The pretreated structure’s aluminum and silicon grains exhibited a preferred orientation, which was slightly mitigated after the HHIP treatment.

## 4. Conclusions

An innovative hydrothermal HIPing (HHIP) approach was hypothesized and tested. The aluminum-based metal, which, according to thermodynamics, at supercritical water conditions of 25 MPa and 300–400 °C should have been totally oxidized, was practically inert at the proposed HHIPing conditions of 300–350 MPa and 250–350 °C applied in this work; this enabled the full HHIP treatment of 6–24 h at these conditions, possibly owing to development of a resistant Al/O native oxides layer. Applying the proposed treatment to AM AlSi10Mg samples, which may be regarded as an aluminum structure that is not easily deformed, resulted in an 85.7% reduction in AM pores and a reduction of 90.8% in the pore surface area at a temperature of 350 °C, which is much lower than the common 500–520 °C applied in argon-based HIPing treatments. The AM AlSi10Mg maintained its original microstructure after 24 h, the longest HHIPing period applied in this work. The treatment, therefore, has the potential to yield better mechanical properties without the deleterious effect of grain growth or Si phase dissolution. The well-known aluminum micro-composite structure was also preserved after 24 h of treatment at 350 °C. This proof of concept may pave the way for further work on different treatment conditions, mechanical properties, surface treatments and modelling the behavior of aluminum alloys, as well as other AM metal parts.

## Figures and Tables

**Table 1:** Metal samples’ composition.

	Al6061 [24]	Al2024 [25]	AlSi10Mg [26]
Aluminum	Balance	Balance	Balance
Manganese	0.15%	0.30–0.90%	0.45%
Silicon	0.40–0.8%	0.50%	9.00–11.00%
Copper	0.15–0.40%	3.80–4.20%	0.05%
Magnesium	0.80–1.20%	1.20–1.80%	0.25–0.45%
Zirconium	0.25%	0.25%	
Zinc			0.10%
Iron	0.70%	0.50%	0.55%
Titanium	0.15%	0.15%	0.15%
Chromium	0.04–0.35%		
Nickel			0.05%
Lead			0.05%
Tin			0.05%
Others each	0.05%	0.05%	
Others total	0.15%	0.15%	

**Table 2:** Experimental conditions, sample weights, and ICP results in the surrounding water samples.

	Material	Temp	Pressure	Time	Initial Weight	Final Weight	Weight Change	Weight Loss	The Main Species in the Sample
#		[°C]	[MPa]	[h]	[g]	[g]	[%]		[mg/L]
1	Water blank	300	300	8				0.013 µm ^(1)^	[Fe] = 53.4, [Ti] = n.d.
2	Al6061	300	300	7	1.4428	1.4450	+0.15	^(2)^	[Al] < 0.1, [Fe] = 21.2, [Ti] = n.d.
3	Al6061	350	300	7	1.5138	1.5183	+0.30	0.15%	[Al] = 0.242, [Fe] = 17.99, [Mg] = 5.1, [Ti] = n.d.
4	Al2024	300	300	7	0.3240	0.3249	+0.28	0.62%	[Al] = 0.085, [Fe] = 12.5 [Mg] = 6.7, [Ti] = n.d.
5	Al2024	350	300	7	0.3952	0.3968	+0.40	0.09%	[Al] < 0.1, [Fe] = 4.8 [Mg] = 1.2, [Ti] = n.d.
6	AlSi10Mg	300	300	6	0.5517	0.5534	+0.31	0.29%	[Al] = 0.200, [Fe] = 8.6, [Si] = 13.2, [Mg] = 1.4, [Ti] = n.d.
7	AlSi10Mg	350	300	6	0.5863	0.5879	+0.27	0.37%	[Al] = 0.289, [Fe] = 14.1, [Si] = 19.9, [Mg] = 1.9, [Ti] = n.d.
8	AlSi10Mg	250	350	6	0.5310	0.5353	+0.81	0.21%	[Al] = 0.278, [Fe] = 33.1, [Si] = 7.8, [Mg] = 1.0, [Ti] = n.d.
9	AlSi10Mg	250	350	24	0.6771	0.6771	none	0.15%	[Al] = 0.129, [Fe] = 37.1, [Si] = 7.8, [Mg] = 0.9, [Ti] = n.d.
10	AlSi10Mg	350	300	24	0.4887	0.4895	+0.16	0.53%	[Al] = 0.045, [Fe] = 21.7, [Si] = 18.4, [Mg] = 2.3, [Ti] = n.d.

^(1)^ Equivalent reactor wall thickness loss. ^(2)^ No data for [Mg].

**Table 3:** Results of microCT tests on the AM AlSi10Mg samples.

Sample	Reference Untreated	Experiment 8	Experiment 9	Experiment 10
Experimental setup	none	250 °C, 350 MPa, 6 h	250 °C, 350 MPa, 24 h	350 °C, 300 MPa, 24 h
Sample volume [mm^3^]	88.7	66.82	77.12	68.99
N_T_ [Voxel/mm^3^] ^(1),(2)^	1807.0	1400.2	832	258.2
Change in N_T_		−22.5%	−54%	−85.7%
S_T_ [mm^2^/mm^3^] × 10^−3^	37.9	23.9	12.2	3.5
Change in S_T_^(3)^		−36.8%	−67.8%	−90.8%
Max diameter [µm]	170	107	112	97

^(1)^ Voxel size = 3.5 µm, ^(2)^ Total pores, ^(3)^ Total pore projected surface.

**Table 4:** AM AlSi10Mg sample hardness.

Experiment	Experiment Setup	HV0.1 Hardness
Reference Untreated	none	134 ± 8
Experiment 7	350 °C, 300 MPa, 6 h	148 ± 25
Experiment 8	250 °C, 350 MPa, 6 h	148 ± 24
Experiment 9	250 °C, 350 MPa, 24 h	140 ± 26
Experiment 10	350 °C, 300 MPa, 24 h	152 ± 28

## Data Availability

Data are contained within the article.
